# Two birds with one stone: can [68Ga]Ga-DOTANOC PET/CT image quality be improved through BMI-adjusted injected activity without increasing acquisition times?

**DOI:** 10.1259/bjr.20211152

**Published:** 2022-03-14

**Authors:** Lucia Zanoni, Diletta Calabrò, Emilia Fortunati, Giulia Argalia, Claudio Malizia, Vincenzo Allegri, Simona Civollani, Stefano Fanti, Valentina Ambrosini

**Affiliations:** Nuclear Medicine Unit, IRCCS Azienda Ospedaliero-Universitaria di Bologna, Bologna, Italy; Department of Experimental Diagnostic and Specialized Medicine (DIMES), Alma Mater Studiorum University of Bologna, Bologna, Italy; Department of Experimental Diagnostic and Specialized Medicine (DIMES), Alma Mater Studiorum University of Bologna, Bologna, Italy; Department of Experimental Diagnostic and Specialized Medicine (DIMES), Alma Mater Studiorum University of Bologna, Bologna, Italy; Nuclear Medicine Unit, IRCCS Azienda Ospedaliero-Universitaria di Bologna, Bologna, Italy; Nuclear Medicine Unit, IRCCS Azienda Ospedaliero-Universitaria di Bologna, Bologna, Italy; Nuclear Medicine Unit, IRCCS Azienda Ospedaliero-Universitaria di Bologna, Bologna, Italy; Nuclear Medicine Unit, IRCCS Azienda Ospedaliero-Universitaria di Bologna, Bologna, Italy; Department of Experimental Diagnostic and Specialized Medicine (DIMES), Alma Mater Studiorum University of Bologna, Bologna, Italy; Nuclear Medicine Unit, IRCCS Azienda Ospedaliero-Universitaria di Bologna, Bologna, Italy; Department of Experimental Diagnostic and Specialized Medicine (DIMES), Alma Mater Studiorum University of Bologna, Bologna, Italy

## Abstract

**Objectives:**

To assess how patients’ dependent parameters may affect [68Ga]Ga-DOTANOC image quality and to propose a theoretical body mass index (BMI)-adjusted injected activity (IA) scheme, to improve imaging of high weight patients.

**Methods:**

Among patients prospectively enrolled (June-2019 and May-2020) in an Institutional Ethical Committee-approved electronic archive, we included those affected by primary gastro-entero-pancreatic (GEP) or lung neuroendocrine tumour and referred by our Institutional clinicians (excluding even minimal radiopharmaceutical extravasation, movement artefacts, renal insufficiency). All PET/CT images were acquired following EANM guidelines and rated for visual quality (1 = non-diagnostic, 2 = poor, 3 = moderate, 4 = good). Collected data included patient’s body mass, height, BMI, age, IA (injected activity), IA/Kg (IAkg), IA/BMI (IABMI), liver SUVmean, liver SUVmax standard deviation, liver-signal-to-noise (LSNR), normalised_LSNR (LSNR_norm) and contrast-to-noise ratio (CNR) for positive scans and were compared to image rating (poor *vs* moderate/good).

**Results:**

Overall, 77 patients were included. Rating concordance was high (agreement = 81.8%, Fleiss k score = 0.806). All patients’ dependent parameters resulted significantly different between poor-rated and moderate/good-rated scans (IA: *p* = 0.006, IAkg: *p* =< 0.001, body weight: *p* =< 0.001, BMI: *p* =< 0.001, IABMI: *p* =< 0.001). Factors significantly associated with moderate/good rating were BMI (*p* =< 0.001), body weight (*p* =< 0.001), IABMI (*p* =< 0.001), IAkg (*p* = 0.001), IA (*p* = 0.003), LSNR_norm (*p* = 0.01). The BMI-based model presented the best predictive efficiency (81.82%). IABMI performance to differentiate moderate/good from poor rating resulted statistically significant (IA-AUC = 0.78; 95% CI: 0.68–0.89; cut-off value of 4.17 MBq*m^2^/kg, sensitivity = 81.1%, specificity = 66.7%). If BMI-adjusted IA (=4.17*BMI) would have been applied in this population, the median IA would have slightly inferior (−4.8%), despite a different IA in each patient.

**Advances in knowledge:**

BMI resulted the best predictor of image quality. The proposed theoretical BMI-adjusted IA scheme (4.17*BMI) should yield images of better quality (especially in high-BMI patients) maintaining practical scanning times (3 min/bed).

## Introduction

Somatostatin receptors PET/CT is the gold standard functional imaging modality^1,2^ for imaging patients with well-differentiated neuroendocrine tumour (NET). Current EANM^1^ guidelines recommend to perform PET/CT for initial staging, restaging after therapy, selection of patients eligible for peptide receptor radionuclide therapy (PRRT) and for the detection of the unknown primary tumour site in patients with proven NET metastatic disease.

Although a wide literature supports the high accuracy of this imaging modality for NET detection,^2,3^ reports potential pitfalls^4,5^ and the impact of PET/CT derived parameters on prognosis,^6–9^ less evidence was published in order to optimise administered dose and image quality. Current EANM guidelines recommend to use a 68Ga-DOTA-peptide (DOTATOC, DOTANOC or DOTATATE) injected activity (IA) ranging from 100 to 200 MBq, also depending on scanners’ characteristics and body weight.^1^ However, a definitive recommendation on the IA to be administered per patient based on body weight is lacking. On the contrary, the FDA recommends to employ a fixed IA of 2 MBq/kg of DOTATATE up to 200 MBq.^10^

In high weight patients, it is well known that image quality decreases (due to increased image noise secondary to the increase in photon attenuation and the scatter fraction) and it is generally recommended to both increase the IA and bed-time acquisition.^11^ However, this would also result in increased scanner occupancy time and higher IA, that together would imply a reduction in the total number of scans acquired per day.

In high volume centres, it is imperative to plan the working schedule in order to optimise the radiopharmaceutical availability, mostly limited by 68Ga-elution from the generator, to achieve the highest number of scans per day. This is even more true since the introduction of novel radiotracers also labelled with 68Ga (*e.g.,* 68Ga-PSMA). Moreover, personalisation of the administered dose is a must in order to reduce unnecessary patients’ radiation exposure,^11^ particularly relevant in the setting of NET patients, considering the relatively long life expectancy after initial diagnosis and consequently the need of multiple PET/CT scans.

The primary aim of the study was to assess how patients’ dependent parameters may affect image quality. Considering the expected relevant role played by BMI, we also assessed whether it is possible to propose a BMI-adjusted IA in order to improve image quality, especially in high weight patients.

## Methods and materials

Among [68Ga]Ga-DOTANOC PET/CT scans of patients prospectively enrolled in an Institutional Ethical commitee-approved (131/2017/O/Oss) electronic archive between June 2019 and May 2020, we consecutively included patients with a primary gastro-entero-pancreatic (GEP) or lung neuroendocrine tumour and referred by our Institutional clinicians and excluded those i) presenting other primary tumour site or unknown primary tumour, ii) even minimal radiopharmaceutical extravasation, movement artefacts or renal insufficiency (for their potential impact on image quality). If more than one scan of the same patient was present, we included only the baseline PET/CT.

Procedures were in accordance with the declaration of Helsinki 2013 and all subjects signed an informed consent form.

In all cases, PET/CT was performed following European Association of Nuclear Medicine (EANM) standard procedure.^1^ In particular, in order to increase IA for higher weight patients, the following institutional protocol was used in routine diagnostic scanning at our centre: a standard injected dose of 100 MBq was administered to patients with body weight below 75 Kg; for higher weight patients, an increase of 0.8 MBq/kg was employed up to a maximum of 200 MBq.

Images were acquired on one of the following GE PET/CT tomographs: two Discovery STE tomographs (100 kV, 120 mA, 0.6 s, 3.75 mm), one Discovery MI (100 kV, 15-200 mA-adjusted, 0.6 s, 3.75 mm) and one Discovery 710 (100 kV, 120 mA, 0.6 s, 3.75 mm). In all cases, images were acquired for 3 min/bed position, arms above the head.

In all cases, collected data included each patient’s body mass (kg), height (cm), BMI (kg/m^2^),^12^ age (year), IA (MBq), IA/kg (IAkg; MBq/kg), IA/BMI (IABMI; MBq*m^2^/Kg).

Patients were classified based on BMI as: underweight (BMI<18.4), normal weight (BMI: 18.5–24.9), pre-obesity (BMI: 25.0–29.9), obesity class I (BMI: 30.0–34.9), obesity class II (BMI: 35.0–39.9), obesity class III (BMI:>40).^12^

Semi-quantitative PET/CT parameters were the liver SUVmean (LSUVmean) and liver SUVmax standard deviation (LSD). The liver signal-to-noise (LSNR) was assessed in a disease-free area of the right liver lobe, using a 2cm-diameter VOI, using the Advantage software GE (VCAR), and was calculated by dividing the LSUVmean (representing liver radiopharmaceutical biodistribution) by the LSD. Normalised LSNR (LSNR_norm) was estimated as follows^13^ : $LSNR\_norm=\frac{LSNR}{\sqrt{\left(IA*bed\;time\;position\right)}}.$

Contrast-to-noise ratio (CNR) was assessed on the primary tumour lesion in positive scans as follows^14^: $CNR=\frac{Lesion\;SUV\;mean-Background\;SUV\;mean}{Background\;SD}$

Images were independently reviewed by three experienced nuclear medicine physicians on [68Ga]Ga-DOTANOC PET/CT images, blinded to clinical data. Reviewers were asked to rate each scan for overall image quality according to a four-point scale (1 = non-diagnostic, 2 = poor, 3 = moderate, 4 = good). For the purposes of the analysis, moderate- and good-rated scans were also grouped as a single category (moderate/good). To define appropriate image quality, a scan had to be rated at least as category three or higher by all reviewers.

Patients’ dependent parameters (body weight, IA, IAkg, BMI, IABMI) were analysed as compared to the image quality rating (poor *vs* moderate/good) and possible predictive factors of image quality were investigated. The performance of the best parameter discriminating image quality was assessed and on optimal cut-off value was calculated.

### Statistical analysis

Anova test or t-test were used to compare differences between patient’s body weight, BMI, IAKg, IABMI on different scanners or reviewer’s ratings (poor *vs* moderate/good) after testing for normality using Kolmogorov-Smirnov test and testing for homogeneity of variances using Bartlett test. The agreement analysis on reviewer’s ratings was performed using the Fleiss’s K coefficient. Mann-Whitney U-test was employed to compare each variable (patient’s body weight, BMI, IAKg, IABMI) between two groups of reviewer’s ratings (poor *vs* moderate/good).

Receiver Operating Characteristics (ROC) curves were performed and areas under the curves (AUC) were calculated for each variable (patient’s body weight, BMI, IAKg, IABMI). Results were reported as estimated value and corresponding 95% confidence interval (CI) for sensitivity, specificity, positive and negative predictive values and AUC.

A logistic regression model was used to evaluate the relationship between reviewers and all variables. A different multivariate logistic regression technique was compared with ANOVA test after multicollinearity assessment. Predictive efficiency was determined by using the confusion matrix 2*2, filled with comparison of predicted observations and true value.

All analyses were performed using R software v. 3.6.1, with package pROC added, and a *p*-value ≤ 0.05 was considered statistically significant.

## Results

Overall, 77 patients were included (median age = 61 (61.3 ± 12.3 [20-86] years). All cases were addressed to PET/CT imaging for characterisation of a pathologically proven GEP or lung NEN. In 44/77 (57%) cases PET/CT images were rated positive while in the remaining cases (33; 43%) there were no areas of tracer pathological uptake.

**Figure 1.**
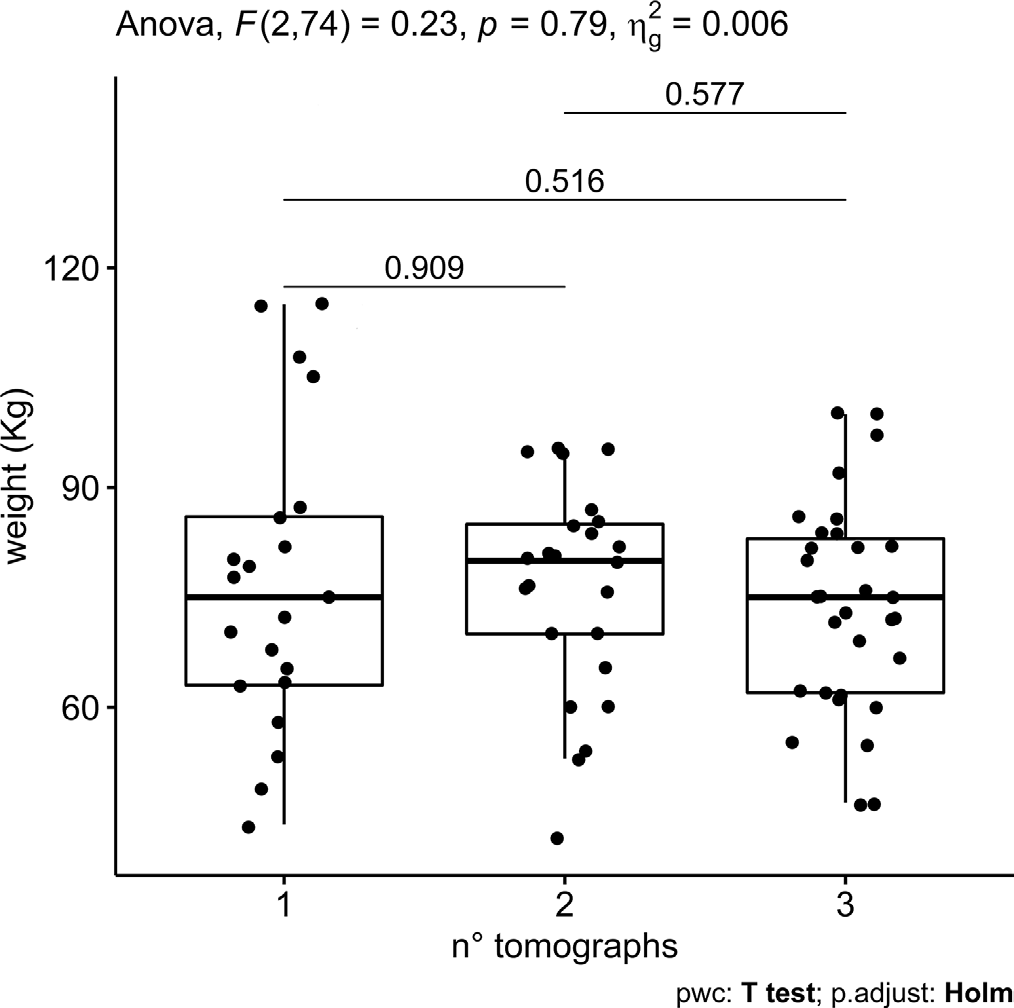
Distribution of patients’ body weight (kg) per Tomograph. Abbreviations: Tomograph 1 = Discovery STE; 2 = Discovery MI; 3 = Discovery 710

**Figure 2.**
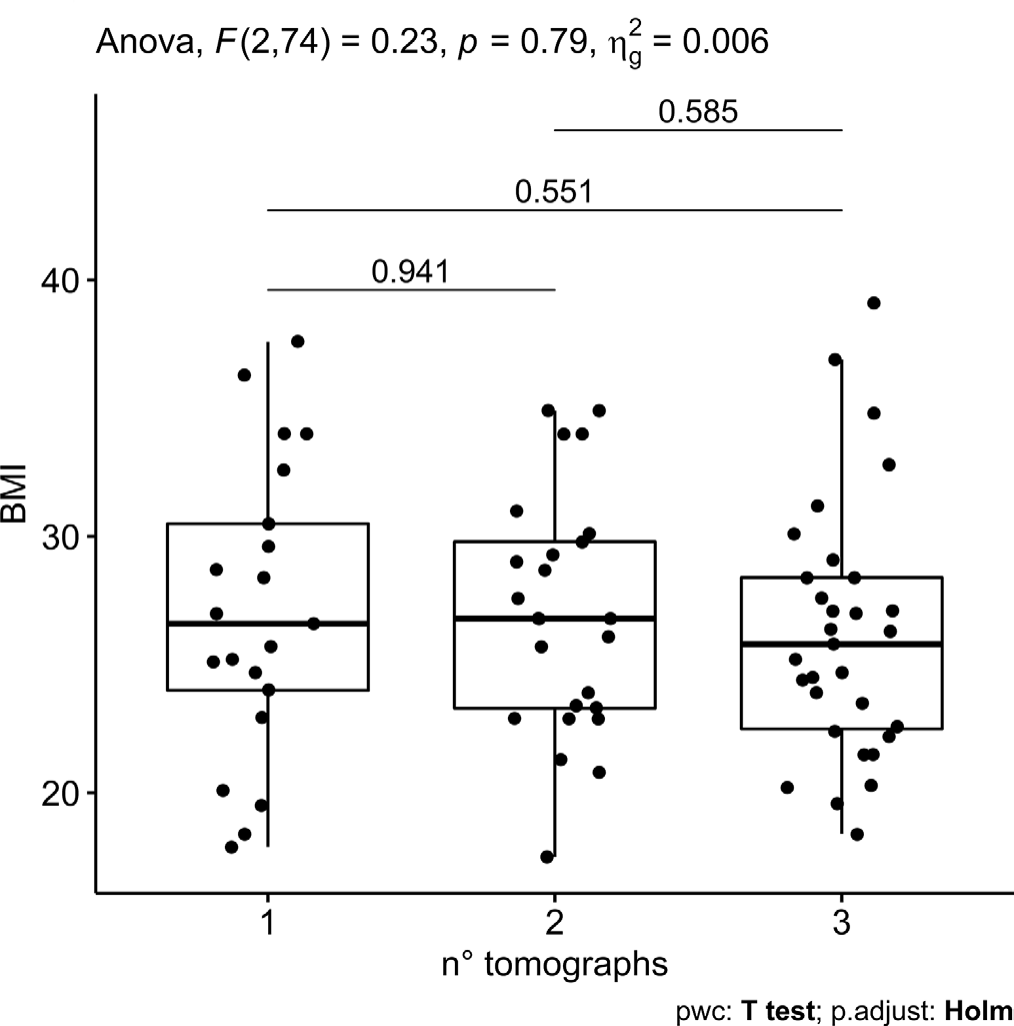
Distribution of patients’ BMI per Tomograph. Abbreviations: BMI = body mass index; Tomograph 1 = Discovery STE; 2 = Discovery MI; 3 = Discovery 710

PET/CT scans were acquired on either Discovery STE (21/77), Discovery MI (25/77), Discovery 710 (31/77). There was no statistically significant difference for patients’ body weight (*p* = 0.77), BMI (*p* = 0.793), IAKg (*p* = 0.969), IABMI (*p* = 0.967), distribution among the three scanners. In particular distribution of patients’ body weight as well of patients’ BMI did not differ significantly between the three different tomographs used for scan acquisition (Figures 1 and 2, respectively).

**Figure 3.**
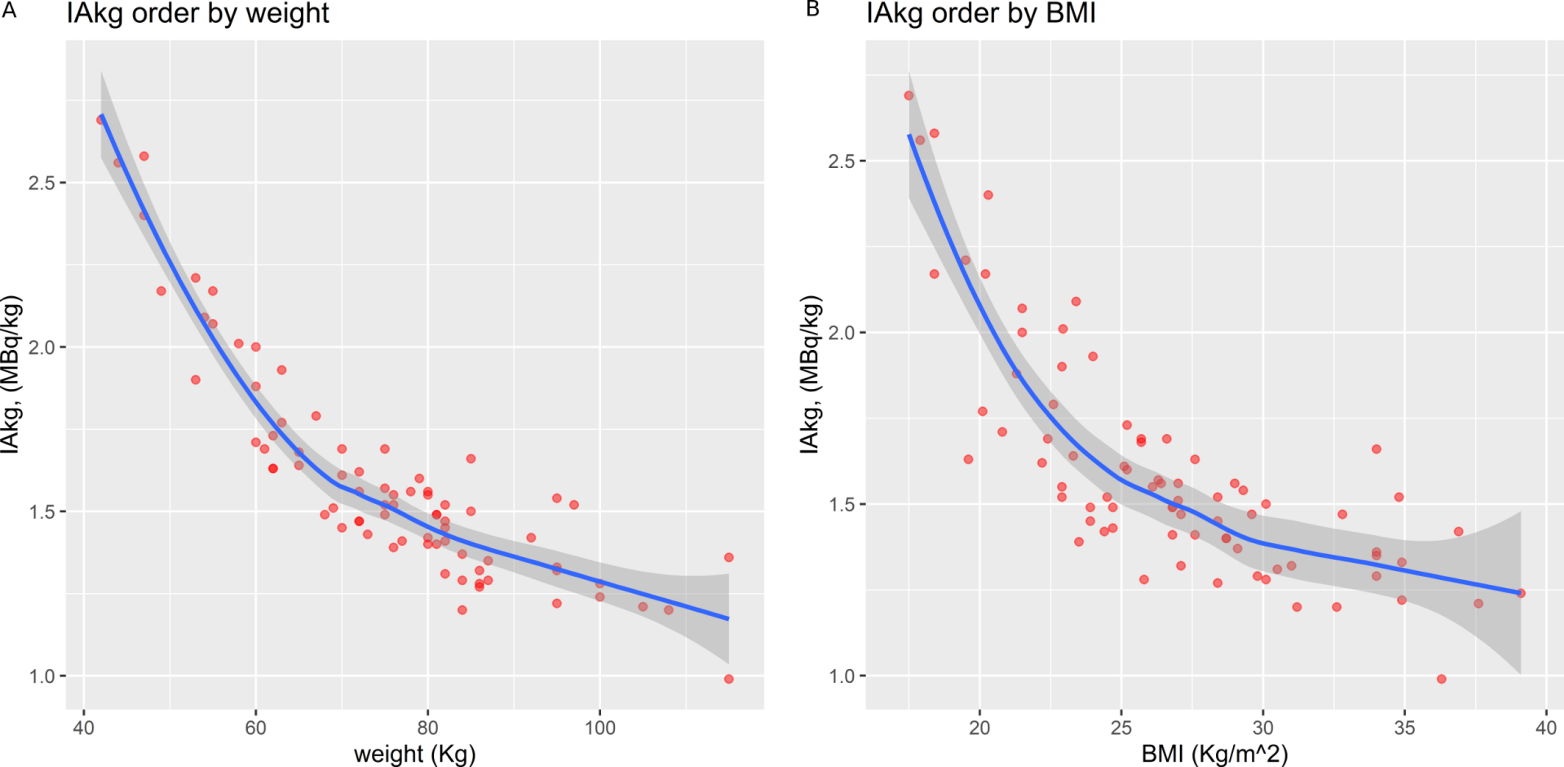
Distribution of IAkg per patients’ body weight (**A**) and per patients’ BMI (**B**).

Patients’ characteristics and PET/CT parameters are summarised in Table 1. Among the studied patients, only 33.7% (26/77) were normal weight and 5.2% (4/77) underweight, while the remaining 47/77 (61%) cases were overweight (pre-obesity: 29/77, 37.7%; obesity class I: 14/77, 18.2%; class II 4/77, 5.2%). The distribution of IAkg/patients’ body weight and per patients’ BMI are presented in Figure 3A and B, respectively.

**Table 1.** Patients' characteristics and PET parameters

	Median	Mean	SD	Min	Max
Weight (kg)	76,0	75,5	15,9	42,0	115,0
Height (cm)	167,0	150,3	52,2	150,0	197,0
BMI	26,4	26,7	5,0	17,5	39,1
Age (years)	61,0	61,3	12,3	20,0	86,0
IA (MBq)	114,2	116,3	10,9	100,6	156,3
IAkg (MBq/Kg)	1,5	1,6	0,3	1,0	2,7
IABMI	4.4	4.5	0.8	3.1	6.6
Uptake time (min)	61,0	62,9	9,7	47,0	98,0
LSUVmean	6,2	6,3	1,6	2,7	10,1
LSD	0,9	0,9	0,3	0,3	2,2
LSNR	6,9	7	1,5	2,4	11,4
LSNR_norm	0,37	0,38	0,09	0,13	0,6
LesionSUVmean	16,9	21	14,8	1,4	68,1
bkSUVmean	2	2,1	1	0,5	4,7
bkSD	0,5	0,6	0,7	0,1	4,7
CNR*	36,8	52,3	53,4	1,9	233

BMI, body mass index; CNR*, contrast-to-noise ratio calculated only for positive scans; IA, injected activity; LSD, liver standard deviation; LSNR, liver signal-to-noise ratio; LSNR_norm, liver signal-to-noise ratio normalised for dose and bed time acquisition; LSUVmean, liver SUVmean; bkSD, background standard deviation; bkSUVmean, background SUVmean.

Reviewers’ ratings of the PET/CT image quality are reported in Table 2. Concordance among reviewers was high (agreement = 81.8%, Fleiss k score = 0.806): the majority of scans was rated as of moderate/good quality (>70%) while no scans resulted non-diagnostic.

**Table 2.** Reviewers' image quality rating (*n* = 77)

Image quality	Reviewer 1		Reviewer 2		Reviewer 3	
Non-diagnostic	0		0		0	
Poor	23	30%	20	26%	17	22%
Moderate	37	48%	36	47%	42	55%
Good	17	22%	21	27%	18	23%
Moderate +good	54	70%	57	74%	60	78%

For the purpose of the following analysis, each scan was considered of appropriate quality only when rated at least as moderate by all reviewers. Patients’ dependent parameters potentially affecting image quality were compared with image quality rating (poor *vs* moderate/good).

**Figure 4.**
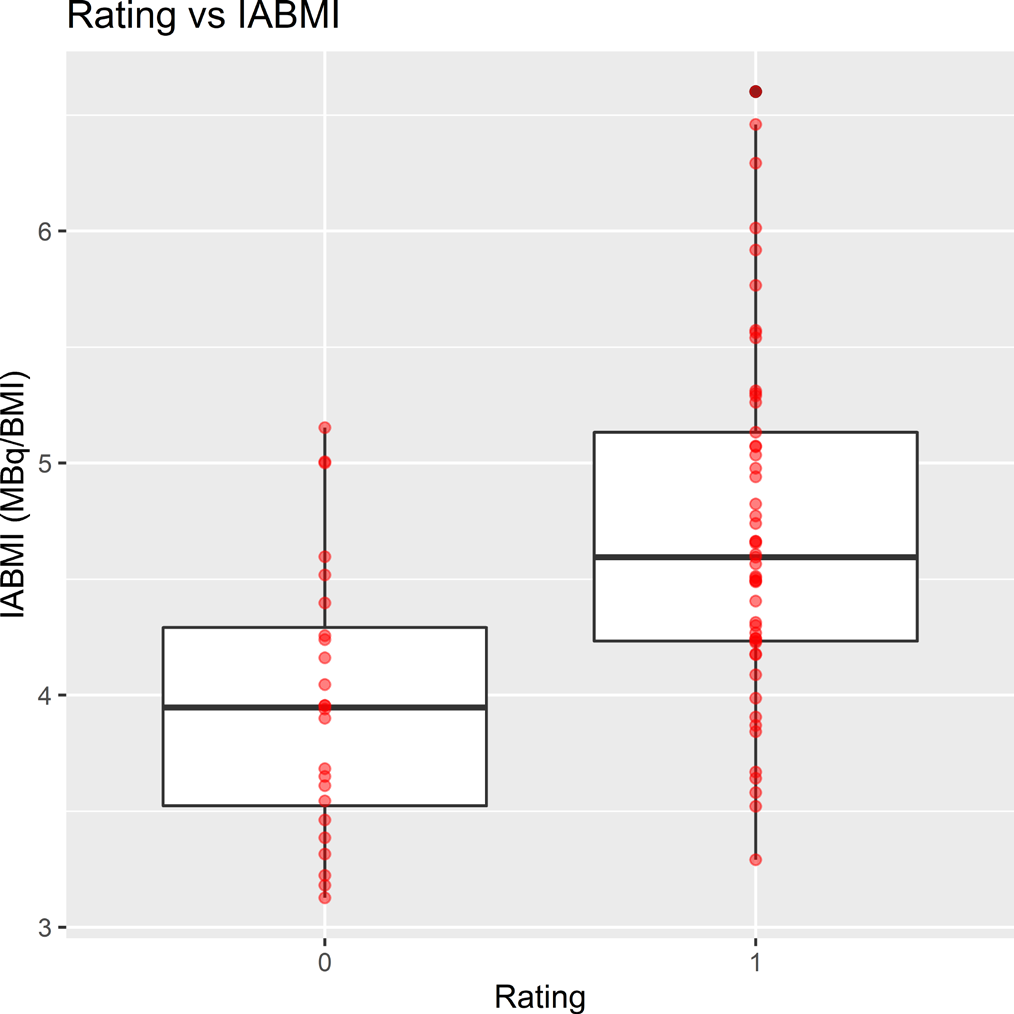
IABMI in poor- and moderate/good-rated scans. Abbreviations: Rating 0 = poor; 1 = moderate/good

**Figure 5.**
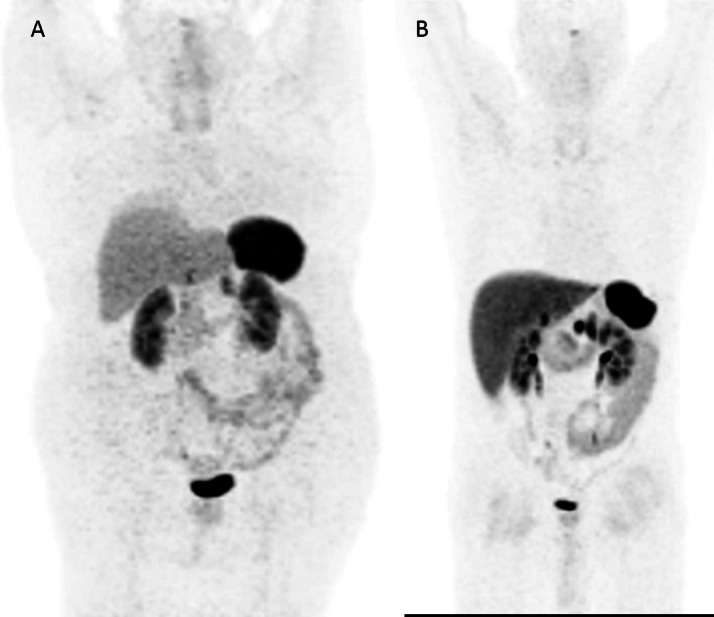
Impact of BMI on visual image quality: higher BMI (39.1) is associated with poor rating (**A**), while lower BMI (23.5) is associated with good rating (**B**).

All patients’ dependent parameters resulted significantly different between scans rated as poor and those rated as moderate/good (IA: *p* = 0.006, IAkg: *p* =< 0.001, body weight: *p* =< 0.001, BMI: *p* =< 0.001, IABMI: *p* =< 0.001). Moderate/good scans were associated with lower body weight and BMI and with higher IAkg and IABMI (Figure 4). On the contrary, poor-rated scans were associated with significantly higher body weight and BMI(Figure 5), in these cases, although a significantly higher IA was administered, lower IAkg and IABMI were observed. Furthermore, even when assessed independently by each reviewer, in moderate/good scans a significantly higher IABMI was observed as compared to poor-rated scans (p reviewer 1 =< 0.001; p reviewer 2 =< 0.001; p reviewer 3 =< 0.001).

LSNR and LSNR_norm were also calculated in all cases: images rated as of moderate/good quality presented a significantly higher mean LSNR value (mean ± sd [range]=7.21±1.65 [2.45–11.41] *vs* 6.50 ± 1.14 [4.20–9.11], *p* = 0.01) and LSNR_norm (mean ± sd [range]=0.39±0.09 [0.13–0.60] *vs* 0.34 ± 0.06 [0.22–0.48]; *p* = 0.001) as compared to images rated as poor. The AUC resulted slightly higher for LSNR_norm (AUC = 0.71, 95% CI 0.6–0.8) as compared to LSNR (AUC = 0.66, 95% CI 0.5–0.8). CNR was calculated in the 44 positive scans; no statistically significant difference was observed between poor and moderate/good scans (*p* = 0.6).

Univariate logistic regression demonstrated that factors significantly associated with moderate/good image rating were BMI (*p* =< 0.001),body weight (*p* =< 0.001), IABMI (*p* =< 0.001), IAkg (*p* = 0.001), IA (*p* = 0.003), LSNR_norm (*p* = 0.016), while body height, age, uptake time and LSNR did not reach statistical significance (Table 3).

**Table 3.** Univariate Logistic Regression Analysis

Predictor	*p*-value	OR	95% CI OR
**Weight (Kg**)	<0.001***	0.89	[0.83, 0.94]
**Height (cm**)	0.708	0.99	[0.98, 1.01]
**BMI**	<0.001 ***	0.67	[0.54, 0.79]
**Age (years**)	0.520	1.01	[0.97, 1.05]
**IA (Mq**)	0.003 **	0.92	[0.86, 0.97]
**IAkg (MBq/Kg**)	0.0014 **	220	[12.2, 9378]
**IABMI**	<0.001 ***	5.65	[2.34, 16.63
**Uptake time(min**)	0.942	1.00	[0.95, 1.05]
**LSUVmean**	0.171	0.80	[0.58, 1.09]
**LSD**	0.128	0.32	[0.07, 1.34]
**LSNR_norm**	0.016 *	4159	[7.43, 7.03*e* + 06]
**LSNR**	0.053	1.41	[1.01, 2.05]
**CNR**	0.279	0.99	[0.98, 1.01]
**Tomographs** (ref. Discovery STE)			
Discovery MI	0.801	0.85	[0.23, 3.01]
Discovery 710	0.778	0.84	[0.24, 2.78]
**Lesion SUVmean**	0.086	0.96	[0.91, 1.003]
**bkSUVmean**	0.404	0.75	[0.37, 1.45]
**bkSD**	0.834	1.11	[0.42, 5.12]

BMI, body mass index; CI, confidence interval; CNR, contrast-to-noise ratio calculated only for positive scans; IA, injected activity; LSD, liver standard deviation; LSNR, liver signal-to-noise ratio; LSNR_norm, liver signal-to-noise ratio normalised for dose and bed time acquisition; LSUVmean, liver SUVmean; OR, odds ratio; bkSD, background standard deviation; bkSUVmean, background SUVmean.

*p ≤ 0.05; **p ≤ 0.01; *p* ≤ 0.001.

The BMI based model presented the best predictive efficiency (81.82%) as compared to the performance of other significant patients’ dependent parameters (body weight = 79.22%; IABMI = 74.03%; IAkg = 72.73%; IA = 71.43%; LSNR_norm = 64.94%).

Multivariate logistic regression was applied after multicollinearity assessment. All multivariate models were not significantly different from each univariate model (Supplementary Table 1).

**Figure 6.**
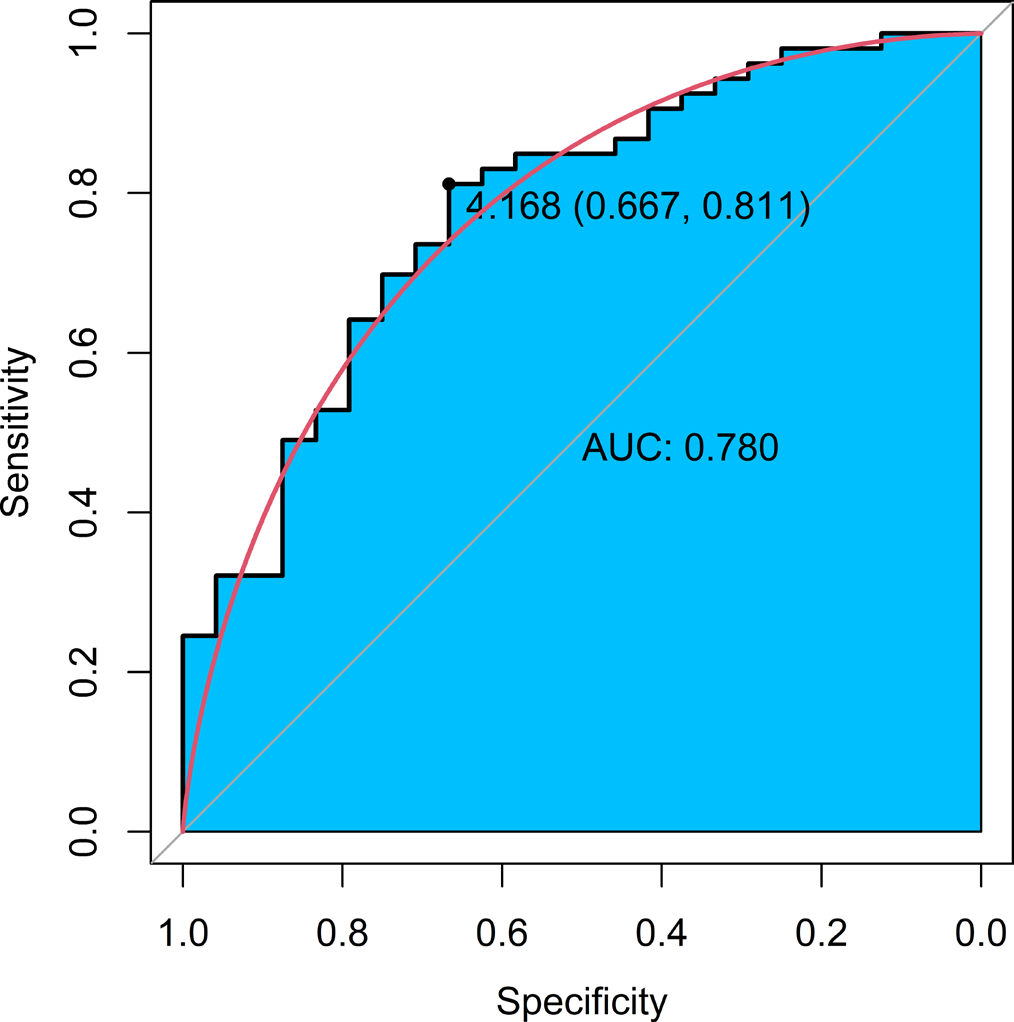
ROC curve and corresponding AUC of IABMI performance to differentiate moderate/good scans versus poor rating. Optimal cut-off value for appropiate quality scans and corresponding sensitivity and specificity.

The higher impact of BMI on image quality as compared to body weight, especially in heavier patients, prompted the need of a BMI-adjusted IA (Figure 4, Figure 5). The performance of IABMI to differentiate moderate/good from poor rating resulted statistically significant (IA-AUC = 0.78; 95% CI: 0.68–0.89): a cut-off value of 4.17 MBq*m^2^/kg allowed to discriminate an appropriate quality scan (sensitivity = 81.1%, specificity = 66.7%, Figure 6).

**Figure 7.**
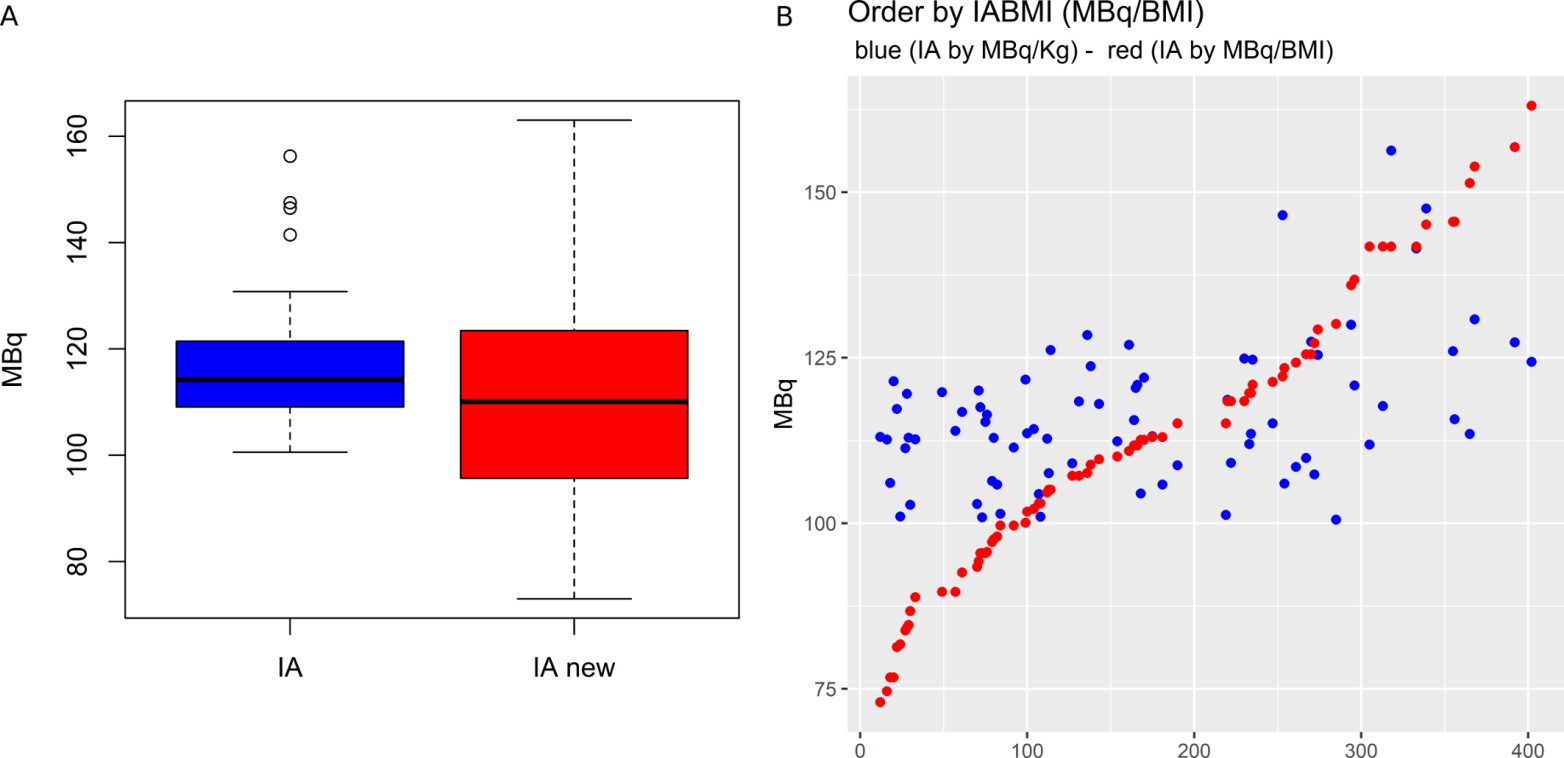
Box-plot representation of the administered IA as compared to the theoretical BMI-adjusted IA (**A**). Point-by-point change in IA (blue dotted line) if the new BMI-adjusted scheme (red dotted line; 4.17*BMI) was implemented (**B**). Abbreviations: IA = injected activity; IAnew = theoretical BMI-adjusted IA

This cut-off value allowed to propose a new scheme for IA calculation, based on a BMI-adjusted IA (BMI-adjusted IA = 4.17*BMI). Figure 7A shows the box-plot difference of the administered IA as compared to the theoretical BMI-adjusted IA and Figure 7B the point-by-point change in IA if the new personalised dose adjustments were implemented. It is worth noticing that if the BMI-adjusted novel scheme would have been applied in this same patients’ population, the median IA would have been almost comparable, slightly inferior (−4.8%) (Table 4), despite a different IA in each patient. In 23/77 (29.9%) patients the IA would have been lower than the one recommended by the EANM guidelines (100 MBq).

**Table 4.** Estimated difference in IA prescribed according to BMI adjustment

	Total	Median	Mean	SD	Min	Max
Administered IA	8955,8	114,2	116,3	17,0	100,6	156,3
Proposed BMI-adjusted IA	8635	111,1	112,2	21,1	73,4	164,1
δ MBq	−320,8	−5,7	−4,2	19,2	−44,3	39,7
δ %	−3,7	−4,8	−6,7	18,6	−57,5	25,6

## Discussion

Several factors can affect PET/CT images quality and most papers investigated their impact on FDG PET/CT images. Factors that might influence image quality include the IA,^15^ the body mass,^16^ the BMI,^17,18^ the time per bed position^19^ and the uptake time.^1^

Tatsumi and colleagues^18^ reported that body habitus affected both statistical/quantitative and qualitative/visual PET image quality in a population of 202 patients (counts in heavy patients were as low as one-fourth those in light patients). Moreover, the distribution of the body weight can influence image quality: patients with larger BMI consistently generated poorer image quality.^20^

Sanchez-Jurado R. et al^17^ proposed a reduction of IA between 9 and 22% by adjusting IA to BMI instead of body weight, while maintaining standard acquisition times and without diminishing diagnostic accuracy. However, current FDG^11^ and 68Ga-DOTA-peptides guidelines^1^ still recommend to administer IA based on patients’ body weight.

Furthermore, current knowledge of factors affecting 68Ga-DOTA-peptides PET/CT image quality is scarce. The present study investigated factors potentially affecting 68Ga-DOTANOC PET/CT image quality in a cohort of 77 NET patients. In our patients population, moderate/good scans were associated with lower IA, body weight and BMI and with higher IAkg and IABMI as compared to poor-rated scans (lower IAkg and IABMI, significantly higher IA). BMI resulted the best predictor of image quality and outperformed all the other patients’ dependent parameters; therefore, we proposed a personalised BMI-adjusted IA scheme to optimise image quality, especially in high weight patients. Moreover, this could also allow to distribute the total available activity among patients of different BMI scanned on the same day. In particular, the proposed scheme adjusts the IA at the two extremes of the BMI curve: lower doses can be used in patients with lower BMI in favour of higher IA for high-BMI patients (still below the upper limit recommended by guidelines).

In the setting of overweight patients, it was demonstrated that prolonging acquisition time per bed position could be more effective than increasing the IA.^21^ In fact, the time per bed position in our study was lower (3 min/bed position) than the one reported in previous reports where scans were rated as good quality by all reviewers only when images were acquired for 6 min/bed position.^13,17^

However, total acquisition times are relevant for daily activities planning in high volume centres. At our centre, approximately 60 to 65 PET/CT scans with several different radiopharmaceuticals are acquired per day, therefore scanning time is a factor to be considered when planning daily schedules. Currently, our four PET/CT tomographs are set to acquire 68Ga-DOTANOC scans at 3 min/bed position. Doubling the acquisition time per pt would necessarily reduce the total number of cases scanned per day, not to mention the patients’ increased discomfort.

Considering the rarity of NET and the availability of 68Ga-DOTApeptides PET/CT across countries,^22^ it is mandatory to optimise the available activity in order to scan as many patients as possible, while maintaining high image quality. This is particulary true for our ENETS (European Neuroendocrine Tumour Society) centre of excellence that represents a reference for patients coming from all over Italy and abroad.

To our knowledge, this is the first study proposing a BMI-adjusted IA scheme for [68Ga]Ga-DOTANOC. Our proposed regimen would allow a median reduction of IA of 4.8% in the whole group, in line with the safety recommendations.^23,24^ Therefore, both goals of image quality and practical scanning times could be achieved (“two birds with one stone”).

The reduction would be of −6% in patients with BMI <25, of −8% in the 25-30BMI subgroup and of −5% in the BMI >30 subgroup. This would imply a better IA distribution over the whole patients group, but at the same time, this would also imply delivering a dose below the lower limit of 100 MBq recommeded by EANM guidelines in most normal BMI patients. This latter issue warrants further validation in larger prospective studies to assess if good image quality would be preserved even with a 6% IA reduction in patients with BMI <25.

It can be argued that acquisition on different tomographs may be considered a bias, however, the distribution of patients’body weight and of BMI were not significantly different among the scanners (and the scanner type resulted is not statistically significant at univariate analysis). We can, therefore, assume that the proposed scheme could be applied in an easy, standardised and reproducible way across different scanners.

One limitation of the current study is that images assessement may be biased by subjective rating; however, it is also to be noted that data obtained by LSNR and LSNR_norm analyses were in line with reviewers’ rating.^25^ Another issue to be considered is the distribution of BMI among the studied patients: there was a relative low prevalence of BMI-obese classes patients, with the majority of cases presenting with either normal BMI (approxiamtely one third) or overweight (approximately 40%).

## Conclusion

In the studied sample, poor-rated scans presented lower IAkg and IABMI notwithstanding a significantly higher IA. BMI resulted the best predictor of image quality. The proposed BMI-adjusted IA (4.17*BMI) should yeld images of better quality (especially in high-BMI patients) while mantaining practical scanning times (3 min/bed position).This also implies the need to further validate in larger future prospective studies if in normal BMI patients the proposed reduction of IA (slightly below the current recommeded lower limit) will preserve image quality.

## Supplementary Material

bjr.20211152.suppl-01
