# Supplementary material for: Two birds with one stone: can [68Ga]Ga-DOTANOC PET/CT image quality be improved through BMI-adjusted injected activity without increasing acquisition times?
Source: Br J Radiol. 2022 Mar 14;95(1134):20211152. doi: 10.1259/bjr.20211152 (PMC10996427; doi:10.1259/bjr.20211152)
Supplement: bjr.20211152.suppl-01 [file bjr.20211152.suppl-01.docx]

**Supplementary TABLE 1. Multivariate Logistic Regression Analysis**

| **Predictor (Model 1)** | **β** | **SE β** | **p-value** | **OR** | **95% CI OR** |
| --- | --- | --- | --- | --- | --- |
| ***Costant*** | 16.771 | 4.711 | **< 0.001 ***** | NA |  |
| ***BMI*** | -0.3907 | 0.1016 | **< 0.001 ***** | 0.67 | [0.53, 0.81] |
| ***IA (Mq)*** | -0.0388 | 0.0347 | 0.2633 | 0.96 | [0.89, 1.02] |
| **Tomographs** (ref. Discovery STE) |  |  |  |  |  |
| Discovery MI | -0.371 | 0.9111 | 0.2183 | 0.69 | [0.11, 4.1] |
| Discovery 710 | -1.105 | 0.8978 | 0.2633 | 0.33 | [0.89, 1.02] |

| **Predictor (Model 3)** | **β** | | **SE β** | **p-value** | **OR** | **95% CI OR** |
| --- | --- | --- | --- | --- | --- | --- |
| ***Costant*** | 14.69 | 4.15 | | **< 0.001 ***** | NA |  |
| ***BMI*** | -0.364 | 0.096 | | **< 0.001 ***** | 0.69 | [0.55, 0.82] |
| ***IA (Mq)*** | -0.032 | 0.032 | | 0.325 | 0.96 | [0.90, 1.03] |

| **Predictor (Model 2)** | **β** | **SE β** | **p-value** | **OR** | **95% CI OR** |
| --- | --- | --- | --- | --- | --- |
| ***Costant*** | 13.085 | 3.040 | **< 0.001 ***** | NA |  |
| ***BMI*** | -0.4203 | 0.0993 | **< 0.001 ***** | 0.66 | [0.52, 0.78] |
| **Tomographs** (ref. Discovery STE) |  |  |  |  |  |
| Discovery MI | -0.531 | 0.8800 | 0.546 | 0.58 | [0.09, 3.21] |
| Discovery 710 | -1.023 | 0.8834 | 0.247 | 0.36 | [0.05, 1.87] |

Legend: SE: standard error; CI: confidence interval; OR: odds ratio; IA: injected activity; BMI: body mass index; ***= p≤0.001; NA=not available.
